# Grading of clear cell renal cell carcinoma by using monoexponential, biexponential, and stretched exponential diffusion-weighted MR imaging

**DOI:** 10.3389/fonc.2024.1456701

**Published:** 2024-10-31

**Authors:** Wenhui Wang, Lingdian Wang, Jing Zhou, Taiyuan Liu, Yan Bai, Meiyun Wang

**Affiliations:** ^1^ Department of Medical Imaging, Zhengzhou University People’s Hospital & Henan Provincial People’s Hospital, Zhengzhou, China; ^2^ Department of Urinary Surgery, Zhengzhou University People’s Hospital & Henan Provincial People’s Hospital, Zhengzhou, China; ^3^ Laboratory of Brain Science and Brain-Like Intelligence Technology, Institute for Integrated Medical Science and Engineering, Henan Academy of Sciences, Zhengzhou, China

**Keywords:** stretched exponential, IVIM, ccRCC, monoexponential, biexponential

## Abstract

**Objectives:**

To evaluate the diagnostic accuracy of monoexponential, biexponential and stretched-exponential diffusion-weighted imaging (DWI) models in the grading of clear cell renal cell carcinoma (ccRCC).

**Materials and Methods:**

Fifty-one patients with pathologically proven ccRCC underwent DWI with fifteen *b* factors (0, 10, 30, 50, 70, 100, 150, 200, 300, 400, 600, 800, 1000, 1500, 2000 sec/mm²) on a 3.0T MR scanner. The isotropic apparent diffusion coefficient (ADC), true diffusion coefficient (ADC_slow_), pseudodiffusion coefficient (ADC_fast_), and fraction of perfusion (f) were derived from DWI using a biexponential model. The water diffusion heterogeneity index (α) and distributed diffusion coefficient (DDC) were derived from DWI using a stretched-exponential model. All values were calculated for the solid area of tumors and compared between high-grade and low-grade ccRCC. The Mann−Whitney U test and receiver operating characteristic (ROC) analysis were used for statistical analysis. The DeLong test was performed to compare the ROC curves.

**Results:**

The mean ADC, DDC, ADC_slow_ and α values were significantly lower in high-grade ccRCC than in low-grade ccRCC (P< 0.01). However, the ADC_fast_ and f were not significantly different between the two groups (P > 0.05). According to the ROC analyses, the AUC for α was 0.941, which was significantly greater than those of the other parameters, with a sensitivity of 100% and a specificity of 84.2%. The DeLong test showed that there were significant differences in the ROCs among ADC_fast_/ADC, ADC_fast_/α, f/ADC_slow_, ADC_fast_/ADC_slow_, f/α, DDC/α, and f/ADC.

**Conclusions:**

Diffusion-related parameters (ADC, DDC, ADC_slow_ and α) could be used to distinguish between low- and high-grade ccRCC. The α derived from the stretched-exponential model may be the most promising parameter for grading ccRCC.

## Introduction

Clear cell renal cell carcinoma (ccRCC) is the most common type of primary malignant epithelial tumor of the kidney and accounts for approximately 70% of all renal cell carcinomas (1). The biological behavior of different grades of ccRCC is heterogeneous. The clinical outcomes of different grades of ccRCC vary. In addition to less traumatic surgeries, such as laparoscopy or partial nephrectomy, radiofrequency ablation can be used to treat low-grade ccRCC. However, high-grade ccRCC has a relatively higher recurrence rate, metastasis rate and mortality. Hence, the assessment of presurgical histologic grade is helpful for determining treatment strategies and evaluating the prognosis. At present, renal biopsy is the most accurate method for characterizing presurgical ccRCC. However, it is invasive and has some risks, such as postsurgical bleeding, infection and biopsy failure. Therefore, a noninvasive and accurate method for the characterization of presurgical ccRCC grades is needed.

Diffusion-weighted imaging (DWI) is a useful noninvasive technique for exploring biological microstructures. The isotropic apparent diffusion coefficient (ADC), which is obtained from DWI with a monoexponential model, has been widely used for the characterization of ccRCC (2–4). Considering the complexity of the tumor microstructure, water diffusion behavior in tumors is considerably more complicated. Hence, advanced fitting models of DWI, including biexponential and stretched exponential models, may provide real-world information on the diffusion behavior of water molecules within tumors (5–9). The biexponential model proposed by Le Bihan et al. (10) can theoretically separate the molecular diffusion coefficient from perfusion. The biexponential model parameters included the true diffusivity (ADC_slow_), which reflects the pure diffusion level; the pseudodiffusion coefficient (ADC_fast_), which is related to the microvascular compartment; and the perfusion fraction (f), which provides a measure of the fractional volume of capillary blood flowing in each voxel. Furthermore, the tumour tissue has a relatively higher cell density and comprises complex microstructure, which restricts the diffusion of water molecules and leads to a non-Gaussian distribution. To tackle the limitation of the hypothesis of two diffusion compartments, as one of the most popular non-Gaussian DWI models, the stretched exponential DWI model, introduced by Bennett et al. (11), is an alternative method that can quantify both tissue heterogeneity and diffusion simultaneously. Then, two new parameters were derived. These parameters were the distributed diffusion coefficient (DDC) and water molecular diffusion heterogeneity index (α). The stretched exponential DWI model has been applied in very few clinical studies thus far. Although some previous studies have shown the diagnostic value of intravoxel incoherent motion (IVIM) imaging in grading ccRCC, they have generally used one or two models, such as the monoexponential and/or biexponential models, and not the stretched-exponential model (2, 6, 7, 12, 13). Hence, the aim of our study was to systematically compare the value of the parameters obtained from three signal attenuation models (monoexponential, biexponential and stretched exponential) for predicting the pathological grade of ccRCC.

## Materials and methods

### Study population

This study protocol was approved by the Institutional Review Board. Written informed consent was obtained from all patients prior to the examination. Seventy-three patients with clinically suspected renal tumors were enrolled in the study from February 2023 to April 2024. The inclusion criteria were as follows: (1) MR imaging was performed in patients prior to the treatment of ccRCC, (2) surgery was performed within two weeks after MRI examination, and (3) a histopathological diagnosis was made according to the Fuhrman nuclear grading system. These tumors were then divided into low-grade (I+II) and high-grade (III+IV) tumors. The exclusion criteria were as follows: (1) MR data were not available owing to the presence of obvious artifacts, (2) antitumor therapy or biopsy was performed before MRI, (3) no dynamic contrast-enhanced (DCE) data were available, and (4) the solid tumor component was unavailable for analysis due to its small size (< 20 mm²). Finally, fifty-one patients were included in this study.

### Magnetic resonance examination

Magnetic resonance imaging was performed in all patients with a 3.0-T whole-body MRI unit (Discovery MR 750; General Electric Medical Systems, Milwaukee, Wisconsin) using an 8-channel phased-array coil (GE Medical System). Conventional MR imaging was performed with the following sequences: breath-hold (in expiration) axial T1-weighted (FSPGR imaging sequence; repetition time (TR)/echo time (TE), 180/2.1 ms; field of view (FOV), 360×360 mm²); axial respiratory triggering T2-weighted (FRFSE imaging sequence; TR/TE, 8000/81.4 ms; FOV 420×420 mm²; slice thickness/gap, 5/1 mm); and Cor respiratory triggering T2-weighted (fat-suppressed; TR/TE, 9230.77/81.4 ms; FOV 420 × 420 mm²; slice thickness/gap, 5/1 mm). Transverse respiratory-triggering DW images were obtained before the administration of contrast agents using the following imaging parameters: single-shot spin−echo echo-planar imaging (EPI); spectral fat saturation; TR/TE, 7058.82/75.6 ms; FOV, 360 × 360 mm²; slice thickness/gap, 5/1 mm; fifteen b values (0, 10, 30, 50, 70, 100, 150, 200, 300, 400, 600, 800, 1000, 1500, and 2000 sec/mm²) (NEX 1 for b = 0-800 sec/mm², NEX 2 for b = 1000-2000 sec/mm²); and a total measurement time of 6 minutes, 14 seconds. Axial T1-weighted sequences were repeated after the injection of gadopentetate dimeglumine (Magnevist; Bayer Schering Pharma, Berlin, Germany).

### Image data analysis and processing

The DW images were transferred to a workstation for processing (Advantage Windows 4.5; GE Medical System). The images were independently processed by two radiologists (with 8 and 10 years of renal MR imaging experience) who were blinded to the histopathology results.

The monoexponential calculation of the ADC using the least-squares model for the linear regression of the logarithmically normalized signals of all b-values was performed as follows (10):


S(b)=S(0) exp(-b ADC),

where S(0) corresponds to the signal without diffusion sensitization, and S(b) corresponds to the signal with diffusion weighting.

Biexponential fitting to the IVIM model separating the vascular compartment and tissue compartment in a DWI measurement was performed according to Le Bihan et al. (10):


$$\text{S}\left(\text{b}\right)=\text{S}\left(0\right)\cdot \text{[f}\cdot \text{exp}\left(-{\text{b ADC}}_{\text{fast}}\right)+\left(1-\text{f}\right)\cdot \text{exp(}-{\text{b}\cdot \text{ADC}}_{\text{slow}}\text{)]}$$

This model assumes 2 compartments: (1) microperfusion, occupying an f of the tissue volume in each voxel and showing an ADC_fast_, and (2) real diffusion, occupying the remaining volume fraction (1-f) and showing an ADC_slow_.

The stretched-exponential model is described as follows (11):


$$\text{S}\left(\text{b}\right)=\text{S}\left(0\right)\cdot \text{exp}\left[-{\left(\text{b DDC}\right)}^{\text{α}}\right],$$

where parameter α varies between 0 and 1, representing the intravoxel water molecular diffusion heterogeneity. By inspection of the equation, an α near 1 indicates that this model approaches monoexponential decay. The DDC is the distributed diffusion coefficient, representing the mean intravoxel diffusion rate.

### ROI positioning and statistical analysis

Two blinded radiologists analyzed all the images independently. Free-hand regions of interest (ROIs) were placed at a solid area of the tumor on the largest slice with reference to T1-enhanced images and T2-weighted images. Areas of necrosis, cysts, hemorrhage, calcifications, perirenal fat invasion, and renal vein and/or inferior cava vein invasion were avoided to ensure more accurate measurements. Then, the selected ROIs were copied to the maps of all the other parameters from the same patient. The median value of each parameter within the ROI was used for statistical analysis. The ROI areas varied from 30 to 120 mm².

All the statistical analyses were performed with SPSS software (version 17.0, SPSS Inc., Chicago). The Mann−Whitney U test was used for comparisons of each parameter between high-grade and low-grade ccRCC. Correlations between all parameters of both models and the Fuhrman nuclear grade were analyzed using Spearman’s rank correlation. To characterize the accuracy of the different parameters, univariate ROC analyses were implemented using MedCalc version 11.3.3.0 (MedCalc Software, Mariakerke, Belgium), and the areas under the curve (AUCs) were compared by using the method developed by DeLong et al. The maximum Youden index was used to determine the optimal sensitivity and specificity, as well as the corresponding cutoff values. Statistical significance was defined as P<0.05.

### Histologic analysis

All histopathological results were available based on surgically resected specimens. A uropathologist (with 12 years of experience in uropathology) who was blinded to the MRI findings reviewed the histological slides. According to the Fuhrman classification system (14), all tumors were subcategorized into ‘‘low-grade’’ (Fuhrman I–II) and ‘‘high-grade’’ groups (Fuhrman III–IV) (12, 15, 16).

## Results

### Study group

In total, 51 ccRCC patients were enrolled in this study. The median age of the 51 enrolled patients was 59 years (range, 33–74 years; 29 males and 22 females). The mean tumor diameter was 4.3 cm (range, 2.7–6.9 cm). Based on the Fuhrman classification system, the patients were divided into four groups: 18 with grade I tumors, 20 with grade II tumors, 10 with grade III tumors, and 3 with grade IV tumors. Due to the small number of patients with Fuhrman grade IV tumors, we classified grades I and II as low grade, while grades III and IV were classified as high grade.

### Associations between diffusion parameters and pathologic diagnoses

**Figure 1:**
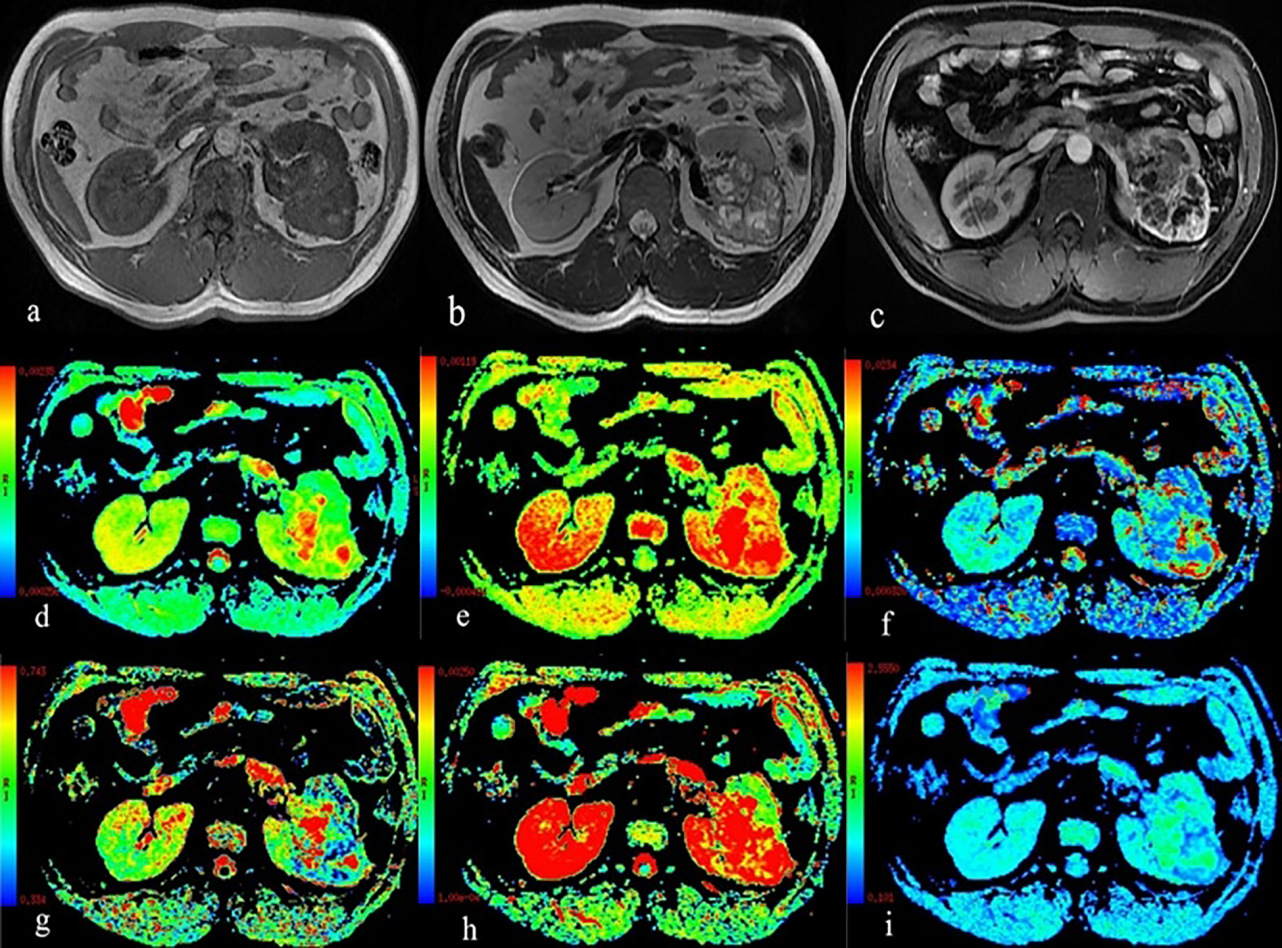
A 59-year-old man with Fuhrman grade III clear cell renal cell carcinoma in the left kidney. **(A)** T1WI map; **(B)** T2WI map; **(C)** Postgadolinium T1WI map; **(D)** the ADC map; **(E)** the ADC_slow_ map; **(F)** the ADCfast map; **(G)** the f map; **(H)** the DDC map; and **(I)** the α map.

**Figure 2:**
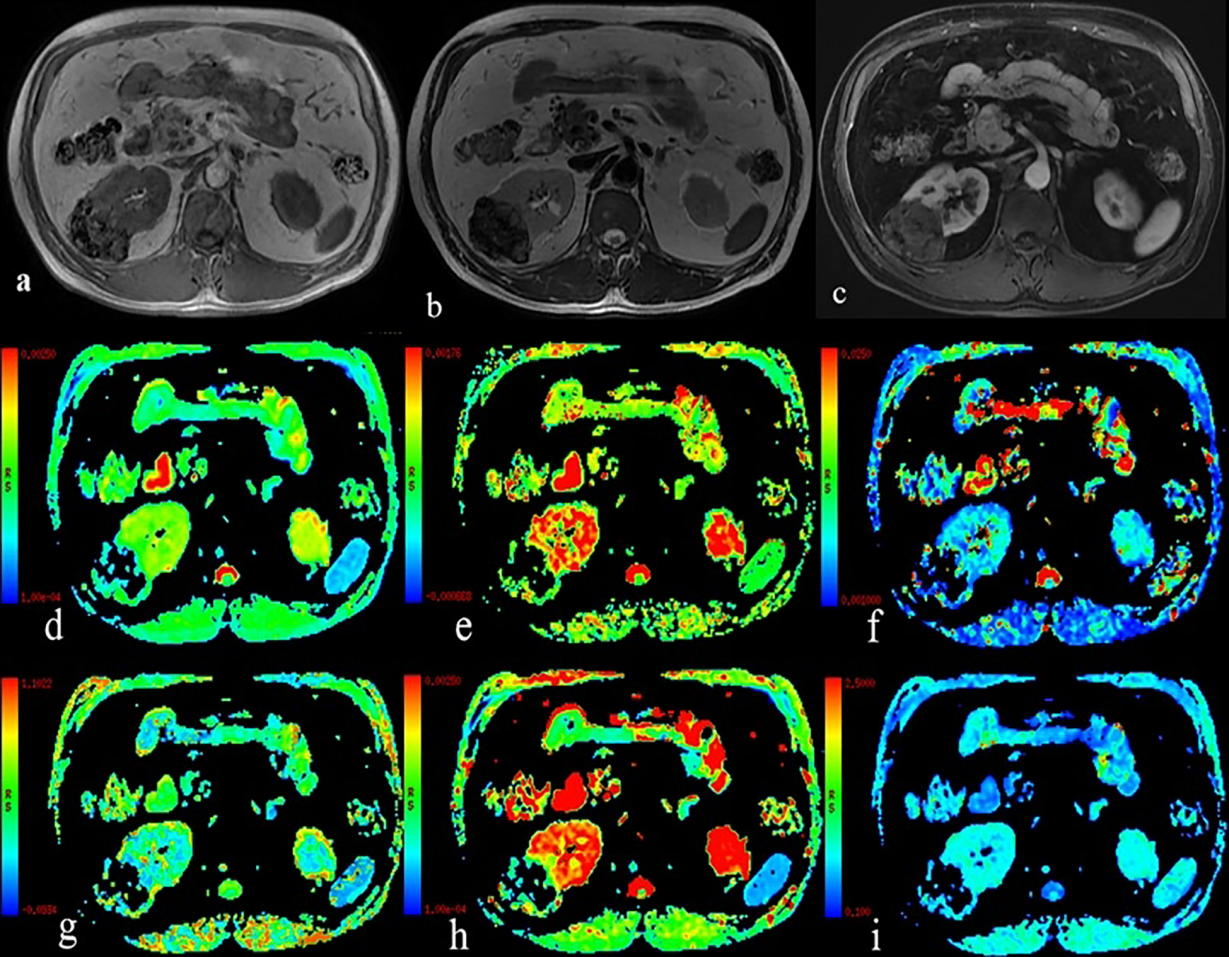
A 54-year-old man with Fuhrman grade II clear cell renal cell carcinoma in the left kidney. **(A)** T1WI map; **(B)** T2WI map; **(C)** Postgadolinium T1WI map; **(D)** the ADC map; **(E)** the ADC_slow_ map; **(F)** the ADC_fast_ map; **(G)** the f map; **(H)** the DDC map; and **(I)** the α map.

Mann–Whitney U tests revealed that the ADC, ADC_slow_, α, and DDC differed significantly between the low-grade and high-grade groups (all Ps< 0.05). No significant difference was found between the two groups for f and ADC_fast_ (p > 0.05). The average ADC, ADC_slow_, α, and DDC values decreased with increasing ccRCC grade (**Table 1**, **Figures 1**, **2**). We found a strong negative correlation between several parameters and the Fuhrman nuclear grade of tumors (including ADC, ADC_slow_, α, and DDC) (**Table 2**). The correlation coefficient of α was -0.666 (P< 0.001). Compared with the other parameters, α had the closest correlation with the Fuhrman nuclear grade.

**Table 1:** Monoexponential, biexponential and stretched-exponential Diffusion Parameters of Low- and High-Grade ccRCCs.

Parameters	Low Grade ccRCCs	High Grade ccRCCs	p Value
ADC	1.28 ± 0.34	0.86 ± 0.21	<0.001
ADC_slow_	1.65 ± 0.25	1.28 ± 0.33	<0.001
ADC_fast_	92.4 ± 50.6	81.4 ± 59.1	0.331
f	0.45 ± 0.17	0.51 ± 0.09	0.275
DDC	2.30 ± 0.71	1.65 ± 0.81	<0.01
α	0.62 ± 0.07	0.48 ± 0.02	<0.001

ADC, ADC_slow_, ADC_fast_, DDC are in units of x10^-3^ mm²/s.

**Table 2:** Correlation of monoexponential, biexponential and stretched-exponential DWI parameters with the Fuhrman nuclear grading of tumors.

	ADC	ADC_slow_	ADC_fast_	f	DDC	α
r	−0.549	−0.474	−0.138	0.154	−0.365	−0.666
P	<0.001	<0.001	0.336	0.279	0.008	<0.001

**Figure 3:**
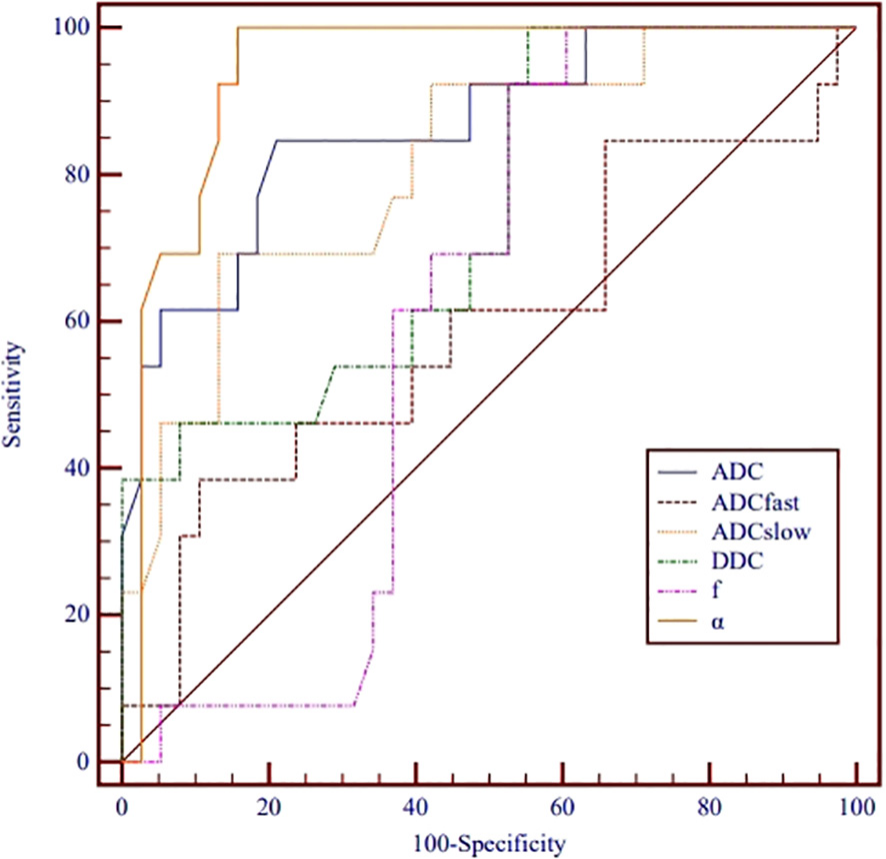
Receiver operating characteristic curves for monoexponential, biexponential and stretched-exponential DWI parameters in distinguishing high- from low-grade ccRCC.

ROC analysis of the utility of the ADC, ADC_slow_, α, and DDC for distinguishing between different nuclear grades is presented in **Table 3** and **Figure 3**. The AUCs for ADC, ADC_slow_, α, and DDC were 0.863, 0.814, 0.941 and 0.742, respectively. The AUC of α was greater than that of the other parameters. The best cutoff point was 0.538x10^-3^ mm²/s, and the sensitivity and specificity were 100% and 84.2%, respectively.

**Table 3:** AUC analysis of DWI parameters and corresponding sensitivity, specificity, and accuracy in prediction of Fuhrman low- and high-grade tumors.

Diffusion Parameter	AUC	95% CI	Cutoff value	Sensitivity (%)	Specificity (%)
α	0.941	0.838-0.988	0.538	100	84.2
DDC	0.742	0.600-0.854	0.0026	100	44.7
ADC_slow_	0.814	0.680-0.909	0.0014	69.2	86.8
ADC_fast_	0.591	0.445-0.727	0.045	38.5	89.5
f	0.602	0.456-0.737	0.396	92.3	47.4
ADC	0.863	0.738-0.943	0.001	84.6	78.9

AUC, area under curve; 95% CI, 95% confidence interval. f and α have no units. Cutoff values are in units of (mm²/s).

**Table 4** shows the comparison of ROC curves using the DeLong test. There were significant differences in the ROC curves between ADC_fast_/ADC_slow_, ADC_fast_/ADC, ADC_fast_/α, f/ADC_slow_, f/ADC, f/α, and DDC/α. (p< 0.05).

**Table 4:** Pairwise comparison of ROC curves using the DeLong test.

	z statistic	p
ADC_fast_/f	0.0946	0.9246
ADC_fast_/ADC_slow_	2.184	0.0290*
ADC_fast_/ADC	2.001	0.0454*
ADC_fast_/DDC	1.222	0.2215
ADC_fast_/α	3.316	0.0009*
f/ADC_slow_	2.342	0.0192*
f/ADC	2.613	0.0090*
f/DDC	1.135	0.2563
f/α	4.214	p< 0.0001
ADC_slow_/ADC	0.491	0.6234
ADC_slow_/DDC	0.690	0.4899
ADC_slow_/α	1.618	0.1057
ADC/DDC	1.497	0.1344
ADC/α	1.067	0.2859
DDC/α	2.207	0.0273*

ROC receiver-operating characteristic. *p< 0.05.

## Discussion

In this study, we compared the diagnostic value of parameters derived from monoexponential, biexponential and stretched-exponential models for differentiating high-grade ccRCC from low-grade ccRCC. Our results showed that the diffusion parameters (ADC, ADC_slow_ and DDC) offered significant clinical value for grading ccRCC. However, the perfusion-related parameters derived from the biexponential model (ADC_fast_ and f) could not be used to discriminate low-grade ccRCC from high-grade ccRCC (p> 0.05). The α derived from the stretched-exponential model had significantly greater diagnostic value than the other diffusion parameters. As such, α may serve as an optimal diffusion parameter for grading ccRCC in clinical practice.

We found that the diffusion parameters (ADC, ADC_slow_ and DDC) were significantly different between high- and low-grade ccRCC (all p< 0.05), which is in line with previous studies (2, 7, 15). This may be due to their histopathological characteristics. It is known that the ADC, ADC_slow_ and DDC values are mainly affected by water molecule diffusion and cell density. High-grade tumors (Fuhrman III–IV) with high nucleus-to-cytoplasm ratios reflect the microenvironment of these lesions, in which the cellular tissue density is greater and the extracellular space is decreased. This further limits the molecular motion of water in these tumor tissues. Therefore, the diffusion parameters (ADC, ADC_slow_ and DDC) can be used to discriminate among ccRCC grades.

In our study, there was no significant difference in the perfusion parameters (ADC_fast_ and f) between high- and low-grade ccRCCs. ADC_fast_ and f, the other two important parameters in the biexponential model, are associated with perfusion and reflect the degree of tissue vascularity without the use of contrast agents. In previous studies, the relationship between perfusion parameters and histological grade has always been controversial (6, 7, 13, 17). The potential reason for this conflicting evidence may be differences in the location of the tumor and the feeding artery. Additionally, variations in the results might be related to the different sample sizes and the number and distribution of b-values in these studies. According to previous studies, due to their intrinsic instability, poor reproducibility, and lower diagnostic efficiency, the ADC_fast_ and f values cannot be used to provide accurate assessments of the tumor grade (18–21). As such, more studies are needed to explore the underlying biological rationale.

In fact, the complexity of biological tissues leads to the restricted diffusion of water molecules, which leads to a non-Gaussian distribution. The stretched exponential model can describe the non-Gaussian behavior of water, which overcomes the limitations of the hypothesis about fast and slow diffusion compartments and the slow exchange between them in a biexponential model. The heterogeneity index α derived from the stretched exponential model is usually thought to reflect tissue heterogeneity and is used to assess tumors. Previous studies have suggested that lower α values indicate greater tissue heterogeneity (9, 22–26). Our current results demonstrated that α was significantly lower in high-grade ccRCC than in low-grade ccRCC. In addition, compared with the other parameters, α had the largest AUC. One possible explanation for our results is that high-grade ccRCC may exhibit more intravoxel diffusion heterogeneity than low-grade ccRCC, which is likely related to a greater degree of histological heterogeneity, microscopic hemorrhage, and tortuous vascular hyperplasia. Thus far, the use of stretched-exponential DWI has limited clinical applicability for the determination of the Fuhrman nuclear grade of ccRCC. Only Zhang Jianjian et al. (17), in their study involving 7 patients with high-grade ccRCC, reported that there was no significant difference in the α parameter between the low- and high-grade tumor groups. This finding was inconsistent with our results, which showed greater negative correlation coefficients between α and the Fuhrman nuclear grade. The possible reasons could be variations in the sample size, equipment, and b-values between the studies. In their study, they used fewer b-values (0, 30, 50, 100, 250, 500, 1000, and 2000 s/mm²) and a relatively smaller sample size of high-grade ccRCC, which may have contributed to the different results between the two studies. Based on our results, α may be a better parameter to use to identify the ccRCC grade.

This study had several limitations. First, the patient population was relatively small. Further studies with a large number of patients are needed to confirm the results. Second, there was a lack of uniform distributed b-sampling to maximize the precision and accuracy of the parameters of the biexponential and stretched-exponential models. Third, the ROIs were selected based on the solid regions on the largest slice instead of the entire renal tumor. This may lead to a certain degree of selection bias owing to the heterogeneity of the tumor. Fourth, the patient cohort was relatively small and we did not evaluate renal tumor histologic subtypes. Chromophobe renal cell carcinoma and papillary renal cell carcinoma are RCC subtypes with overall favorable prognosis if compared with ccRCC. Last, due to the longer DWI time, the images were more susceptible to motion artifacts, particularly those tumors located at the upper or lower pole.

In summary, our study demonstrated that these parameters (ADC, ADC_slow_, DDC and α) can be used to accurately differentiate between low- and high-grade ccRCC, while perfusion-related parameters (ADC_fast_ and f) could not be used to distinguish between high- and low-grade ccRCC. Compared with parameters derived from mono-exponential and biexponential models, the α derived from the stretched-exponential model might be the most promising parameter for evaluating the grade of ccRCC.

## Data Availability

The original contributions presented in the study are included in the article/supplementary material. Further inquiries can be directed to the corresponding author.

## References

[1] Znaor A Lortet-Tieulent J Laversanne M Jemal A Bray F. International variations and trends in renal cell carcinoma incidence and mortality. Eur Urol. (2015) 67:519–30. doi: 10.1016/j.eururo.2014.10.002 25449206

[2] Parada VC Mc CR Miller FH. Can diffusion-weighted magnetic resonance imaging of clear cell renal carcinoma predict low from high nuclear grade tumors. Abdom Radiol (NY). (2017) 42:1241–9. doi: 10.1007/s00261-016-0981-7 27904923

[3] Yu X Lin M Ouyang H Zhou C Zhang H. Application of ADC measurement in characterization of renal cell carcinomas with different pathological types and grades by 3.0T diffusion-weighted MRI. Eur J Radiol. (2012) 81:3061–6. doi: 10.1016/j.ejrad.2012.04.028 22651905

[4] Rosenkrantz AB Niver BE Fitzgerald EF Babb JS Chandarana H Melamed J. Utility of the apparent diffusion coefficient for distinguishing clear cell renal cell carcinoma of low and high nuclear grade. AJR Am J Roentgenol. (2010) 195:W344–51. doi: 10.2214/AJR.10.4688 20966299

[5] Bai Y Lin Y Tian J Shi D Cheng J Haacke E M. Grading of gliomas by using monoexponential, biexponential, and stretched exponential diffusion-weighted MR imaging and diffusion kurtosis MR imaging. Radiology. (2016) 278:496–504. doi: 10.1148/radiol.2015142173 26230975 PMC4734159

[6] Zhu Q Ye J Zhu W Wu J Chen W. Value of intravoxel incoherent motion in assessment of pathological grade of clear cell renal cell carcinoma. Acta Radiol. (2018) 59:121–7. doi: 10.1177/0284185117716702 28648123

[7] Shen L Zhou L Liu X Yang X. Comparison of biexponential and monoexponential DWI in evaluation of Fuhrman grading of clear cell renal cell carcinoma. Diagn Interv Radiol. (2017) 23:100–5. doi: 10.5152/dir.2016.15519 PMC5338574 28050950

[8] Li H Liang L Li A Hu Y Hu D Li Z. Monoexponential, biexponential, and stretched exponential diffusion-weighted imaging models: Quantitative biomarkers for differentiating renal clear cell carcinoma and minimal fat angiomyolipoma. J Magnetic Resonance Imaging. (2017) 46:240–7. doi: 10.1002/jmri.25524 27859853

[9] Wen D Peng P Yue X Xu C Pu Q Ming Y. Comparative study of stretched-exponential and kurtosis models of diffusion-weighted imaging in renal assessment to distinguish patients with primary aldosteronism from healthy controls. PloS One. (2024) 19:e0298207. doi: 10.1371/journal.pone.0298207 38330049 PMC10852313

[10] Le Bihan D Breton E Lallemand D Aubin ML Vignaud J Laval-Jeantet M. Separation of diffusion and perfusion in intravoxel incoherent motion MR imaging. Radiology. (1988) 168:497–505. doi: 10.1148/radiology.168.2.3393671 3393671

[11] Bennett KM Schmainda KM Bennett RT Rowe DB Lu H Hyde JS. Characterization of continuously distributed cortical water diffusion rates with a stretched-exponential model. Magn Reson Med. (2003) 50:727–34. doi: 10.1002/mrm.10581 14523958

[12] Cao J Luo X Zhou Z Duan Y Xiao L Sun X. Comparison of diffusion-weighted imaging mono-exponential mode with diffusion kurtosis imaging for predicting pathological grades of clear cell renal cell carcinoma. Eur J Radiol. (2020) 130:109195. doi: 10.1016/j.ejrad.2020.109195 32763475

[13] Ye J Xu Q Wang S Zheng J Dou W. Quantitative evaluation of intravoxel incoherent motion and diffusion kurtosis imaging in assessment of pathological grade of clear cell renal cell carcinoma. Acad Radiol. (2020) 27:e176–82. doi: 10.1016/j.acra.2019.10.010 31727569

[14] Fuhrman SA Lasky LC Limas C. Prognostic significance of morphologic parameters in renal cell carcinoma. Am J Surg Pathol. (1982) 6:655–63. doi: 10.1097/00000478-198210000-00007 7180965

[15] Goyal A Sharma R Bhalla AS Gamanagatti S Seth A Iyer VK. Diffusion-weighted MRI in renal cell carcinoma: a surrogate marker for predicting nuclear grade and histological subtype. Acta Radiol. (2012) 53:349–58. doi: 10.1258/ar.2011.110415 22496427

[16] Kang SK Zhang A Pandharipande PV Chandarana H Braithwaite RS Littenberg B. DWI for renal mass characterization: systematic review and meta-analysis of diagnostic test performance. AJR Am J Roentgenol. (2015) 205:317–24. doi: 10.2214/AJR.14.13930 26204281

[17] Zhang J Suo S Liu G Zhang S Zhao Z Xu J. Comparison of monoexponential, biexponential, stretched-exponential, and kurtosis models of diffusion-weighted imaging in differentiation of renal solid masses. Korean J Radiol. (2019) 20:791–800. doi: 10.3348/kjr.2018.0474 30993930 PMC6470087

[18] Sun H Xu Y Xu Q Shi K Wang W. Rectal cancer: Short-term reproducibility of intravoxel incoherent motion parameters in 3.0T magnetic resonance imaging. Med (Baltimore). (2017) 96:e6866. doi: 10.1097/MD.0000000000006866 PMC5428618 28489784

[19] Luo M Zhang L Jiang XH Zhang WD. Intravoxel incoherent motion diffusion-weighted imaging: evaluation of the differentiation of solid hepatic lesions. Transl Oncol. (2017) 10:831–8. doi: 10.1016/j.tranon.2017.08.003 PMC5595232 28866259

[20] Andreou A Koh DM Collins DJ Blackledge M Wallace T Leach MO. Measurement reproducibility of perfusion fraction and pseudodiffusion coefficient derived by intravoxel incoherent motion diffusion-weighted MR imaging in normal liver and metastases. Eur Radiol. (2013) 23:428–34. doi: 10.1007/s00330-012-2604-1 23052642

[21] Jerome NP Vidić I Egnell L Sjøbakk TE Østlie A Fjøsne HE. Understanding diffusion-weighted MRI analysis: Repeatability and performance of diffusion models in a benign breast lesion cohort. NMR Biomedicine. (2021) 34:e4508. doi: 10.1002/nbm.v34.7 33738878

[22] Ji Y Xu J Wang Z Guo X Kong D Wang H. Application of advanced diffusion models from diffusion weighted imaging in a large cohort study of breast lesions. BMC Med Imaging. (2023) 23:52. doi: 10.1186/s12880-023-01005-6 37041466 PMC10091641

[23] Wen D Xu C Deng L Yan W Peng P Yue X. Monoexponential, biexponential, stretched-exponential and kurtosis models of diffusion-weighted imaging in kidney assessment: comparison between patients with primary aldosteronism and healthy controls. Abdominal Radiol (New York). (2023) 48:1340–9. doi: 10.1007/s00261-023-03833-0 36745206

[24] Liao D Liu YC Liu JY Wang D Liu XF. Differentiating tumour progression from pseudoprogression in glioblastoma patients: a monoexponential, biexponential, and stretched-exponential model-based DWI study. BMC Med Imaging. (2023) 23:119. doi: 10.1186/s12880-023-01082-7 37697237 PMC10494379

[25] Zheng L Jiang P Lin D Chen X Zhong T Zhang R. Histogram analysis of mono-exponential, bi-exponential and stretched-exponential diffusion-weighted MR imaging in predicting consistency of meningiomas. Cancer Imaging. (2023) 23:117. doi: 10.1186/s40644-023-00633-z 38053183 PMC10696773

[26] Zhao L Liang M Yang Y Zhang H Zhao X. Prediction of false-negative extramural venous invasion in patients with rectal cancer using multiple mathematical models of diffusion-weighted imaging. Eur J Radiol. (2021) 139:109731. doi: 10.1016/j.ejrad.2021.109731 33905979

